# Quantitative assessment of inflammatory infiltrates in kidney transplant biopsies using multiplex tyramide signal amplification and deep learning

**DOI:** 10.1038/s41374-021-00601-w

**Published:** 2021-05-18

**Authors:** Meyke Hermsen, Valery Volk, Jan Hinrich Bräsen, Daan J. Geijs, Wilfried Gwinner, Jesper Kers, Jasper Linmans, Nadine S. Schaadt, Jessica Schmitz, Eric J. Steenbergen, Zaneta Swiderska-Chadaj, Bart Smeets, Luuk B. Hilbrands, Friedrich Feuerhake, Jeroen A. W. M. van der Laak

**Affiliations:** 1 Department of Pathology, Radboud University Medical Center, Nijmegen, The Netherlands; 2 Institute for Pathology, Hannover Medical School, Hannover, Germany; 3 Department of Nephrology, Hannover Medical School, Hannover, Germany; 4 Department of Pathology, Amsterdam University Medical Centers, Amsterdam, The Netherlands; 5 Department of Pathology, Leiden University Medical Center, Leiden, The Netherlands; 6 Center for Analytical Sciences Amsterdam (CASA), Van ‘t Hoff Institute for Molecular Sciences (HIMS), University of Amsterdam, Amsterdam, The Netherlands; 7 Institute of Diagnostic and Interventional Neuroradiology, Hannover Medical School, Hannover, Germany; 8 Faculty of Electrical Engineering, Warsaw University of Technology, Warsaw, Poland; 9 Department of Nephrology, Radboud University Medical Center, Nijmegen, The Netherlands; 10 Institute for Neuropathology, University Clinic Freiburg, Freiburg, Germany; 11 Center for Medical Image Science and Visualization, Linköping University, Linköping, Sweden

**Keywords:** Medical research, Transplant immunology, Kidney

## Abstract

Delayed graft function (DGF) is a strong risk factor for development of interstitial fibrosis and tubular atrophy (IFTA) in kidney transplants. Quantitative assessment of inflammatory infiltrates in kidney biopsies of DGF patients can reveal predictive markers for IFTA development. In this study, we combined multiplex tyramide signal amplification (mTSA) and convolutional neural networks (CNNs) to assess the inflammatory microenvironment in kidney biopsies of DGF patients (*n* = 22) taken at 6 weeks post-transplantation. Patients were stratified for IFTA development (<10% versus ≥10%) from 6 weeks to 6 months post-transplantation, based on histopathological assessment by three kidney pathologists. One mTSA panel was developed for visualization of capillaries, T- and B-lymphocytes and macrophages and a second mTSA panel for T-helper cell and macrophage subsets. The slides were multi spectrally imaged and custom-made python scripts enabled conversion to artificial brightfield whole-slide images (WSI). We used an existing CNN for the detection of lymphocytes with cytoplasmatic staining patterns in immunohistochemistry and developed two new CNNs for the detection of macrophages and nuclear-stained lymphocytes. F1-scores were 0.77 (nuclear-stained lymphocytes), 0.81 (cytoplasmatic-stained lymphocytes), and 0.82 (macrophages) on a test set of artificial brightfield WSI. The CNNs were used to detect inflammatory cells, after which we assessed the peritubular capillary extent, cell density, cell ratios, and cell distance in the two patient groups. In this cohort, distance of macrophages to other immune cells and peritubular capillary extent did not vary significantly at 6 weeks post-transplantation between patient groups. CD163^+^ cell density was higher in patients with ≥10% IFTA development 6 months post-transplantation (*p* < 0.05). CD3^+^CD8^−^/CD3^+^CD8^+^ ratios were higher in patients with <10% IFTA development (*p* < 0.05). We observed a high correlation between CD163^+^ and CD4^+^GATA3^+^ cell density (*R* = 0.74, *p* < 0.001). Our study demonstrates that CNNs can be used to leverage reliable, quantitative results from mTSA-stained, multi spectrally imaged slides of kidney transplant biopsies.

## Abstract (web summary)

This study describes a methodology to assess the microenvironment in sparse tissue samples. Deep learning, multiplex immunohistochemistry, and mathematical image processing techniques were incorporated to quantify lymphocytes, macrophages, and capillaries in kidney transplant biopsies of delayed graft function patients. The quantitative results were used to assess correlations with development of interstitial fibrosis and tubular atrophy.

## Introduction

Delayed graft function (DGF) after kidney transplantation is multifactorial and mainly related to donor characteristics and ischemia time. DGF is generally described as the need for dialysis within 7 days post-transplantation and is a strong risk factor for chronic kidney graft injury [1–3]. A classical component of chronic kidney injury is the presence of interstitial fibrosis and tubular atrophy (IFTA). However, not all DGF patients progress to the development of IFTA and the complex relationship between DGF and IFTA is still poorly understood. This is first due to the lag time between potentially causative events and functional decline, and second because of the variable and complex effects of potential inducers such as rejection and side effects of medication [1, 4]. The general presence of inflammation and specifically macrophages has been described in numerous studies as a predictor for graft loss [5–8]. However, the underlying pathological processes are not fully understood, and high levels of inflammation do not invariably lead to long-term graft loss. As a result of environmental stimuli, macrophages acquire specialized functions and polarize into different phenotypes. Numerous studies suggest that specific macrophage subtypes (alternatively activated macrophages) are involved in tissue remodeling by inducing tissue repair or fibrosis. The polarization toward a tissue remodeling (sometimes pro-fibrotic) phenotype is known to be dependent on a wide range of environmental stimuli, among others provided by T-helper lymphocyte subtypes [9–11]. Assessment of T-helper cell populations in the graft at the time of DGF revealed a prevalent T-helper 1 subtype, but correlations to graft outcome or progression to IFTA were not investigated so far [12]. Comprehensive assessment of the inflammatory microenvironment, specifically focused on macrophages and T-helper cell subsets in carefully selected patient cohorts, might provide insight into why some, but not all DGF patients progress to the development of IFTA.

However, comprehensive investigation of inflammatory infiltrates is hampered by several (technical) limitations. Traditional immunohistochemistry (IHC) and immunofluorescence techniques support visualization of only a limited number of cell markers in one tissue section. Serial sectioning of small, valuable tissue fragments such as kidney biopsies is not desired and the interpretation of relationships between cells in different sections is difficult. In addition, quantitative assessment of the inflammatory infiltrates by visual estimation comes with a significant level of interobserver variability [13]. Traditional image processing techniques such as pixel thresholding, watershed, and morphology-based segmentation rely on prior knowledge of all morphologic cell representations and tissue stain intensity throughout a data set [14–16]. Therefore, these methods often lack robustness for biological and technical image variations and translate poorly to new or external data sets. The rise of digital pathology has accelerated the development of alternative methods for the assessment of whole-slide images (WSI) [17, 18]. Deep learning models, specifically, convolutional neural networks (CNNs) have proven to be capable of segmenting and detecting relevant biological structures in histopathological slides [19–23]. These techniques have the potential to move from subjective visual estimation and traditional image processing to accurate, objective, and reproducible cell detection.

The aim of this study is to develop a method for objective, quantitative assessment of multiple inflammatory cell markers, circumventing the need for extensive serial slide sectioning. To do so, we combine multiplex IHC, multispectral imaging, and deep learning models. To demonstrate the applicability of these techniques, we study the correlations of the inflammatory microenvironment, quantified by deep learning models, with the development of IFTA in surveillance graft biopsies of DGF patients.

## Materials and methods

To assess the inflammatory microenvironment in kidney biopsies of DGF patients, we performed multiplex IHC on surveillance biopsies taken at 6 weeks post-transplantation. Patients were stratified for IFTA development (<10% versus ≥10%) from 6 weeks to 6 months post-transplantation, based on histopathological assessment by three kidney pathologists. Multiplex IHC was performed using tyramide signal amplification (mTSA) panels. One mTSA panel was designed for the visualization of capillaries, macrophages, and T and B lymphocytes (panel I) and one mTSA panel for the visualization of polarized T-helper lymphocytes and macrophages (panel II). Second, the mTSA slides were multi spectrally imaged, and custom-made python scripts were used to convert the multispectral images to artificial brightfield IHC WSI. Converting the slides to artificial IHC WSI allowed for the application of an existing CNN for the detection of lymphocytes in IHC [22]. This existing CNN was designed for cytoplasmatic lymphocyte markers. Hence, a second and third CNN were developed in this study for the quantification of macrophages and nuclear lymphocyte markers in IHC WSI. These three CNNs were subsequently used to quantitatively assess the inflammatory infiltrates in the two patient groups and to study the correlations of the inflammatory microenvironment at 6 weeks post-transplantation with the development of IFTA 6 months after transplantation.

### Tissue samples

We used surveillance biopsies from kidney transplant recipients at Hannover Medical School (Hannover, Germany), acquired in the context of a prospective surveillance biopsy program. Inclusion criteria were: DGF occurrence (defined as <500 ml urine production within the first 24 h after transplantation and/or the need for dialysis within 7 days post-transplantation), absence of rejection in any of the surveillance biopsies or biopsies for cause within the first year post-transplantation, and absence of IFTA in the surveillance biopsy taken at 6 weeks after transplantation (based on the pathology report and graded according to the Banff lesion grading system [24]). All patients were treated with dialysis because of no, or insufficient graft function, variably manifested by (combinations of) anuria, oliguria, metabolic de-arrangement with acidosis or hyperkalaemia. None of the patients had hyperkalaemia or hypervolemia alone. Formalin-fixed, paraffin-embedded tissue (FFPE) from biopsies taken 6 weeks and 6 months post-transplantation was collected. Six patients did not undergo a surveillance biopsy procedure 6 months after transplantation. Instead, the surveillance biopsy taken at 3 months post-transplantation was included (*n* = 3) or the nearest biopsy for cause (*n* = 3, 2.5, 4.3, and 4.6 months post-transplantation). Hereinafter the biopsies are referred to as “6 weeks biopsies” and “6 months biopsies.” If sufficient residual tissue was present in the tissue block for this study, three consecutive slides (2 µm thick) were cut from the 6 weeks biopsy, and one slide from the 6 months biopsy. One slide from both time points was stained using periodic acid-Schiff (PAS) reagent. The remaining two slides from the 6 weeks biopsy were stained using our mTSA panels (see “Multiplex TSA staining” in “Materials and methods”). Cases with sufficient cortical tissue (here defined as ≥4 glomeruli) in both the 6 weeks and the 6 months biopsy were included in the study (*n* = 24). One case was excluded because of interstitial nephritis of unknown cause and one more case due to fixation artifacts. A final number of 22 patients were included in this study (Table 1).

**Table 1:** Patient and donor characteristics categorized by the IFTA development (<10% or ≥10%) from 6 weeks to 6 months post-transplantation.

	ΔIFTA < 10% (*n* = 9)	ΔIFTA ≥ 10% (*n* = 13)
Recipient		
Female (%)	3 (33.3)	7 (53.8)
Age, yr	54.4 (36.7–66.0)	56.8 (32.8–69.3)
BMI, kg/m^2^	27.5 (22.7–31.0)	27.9 (22.2–30.4)
Dialysis time, months	79.4 (7.5–109.3)	57.8 (17.5–196.6)
Pre-formed panel reactive antibodies, %	0 (0–0)	0 (0–85)
Number of transplants	1 (1–1)	1 (1–3)
Underlying renal disease		
Glomerulonephritis/vasculitis	1 (11.1)	3 (23.1)
Tubulo-interstitial disease	1 (11.1)	1 (7.7)
Hypertensive/diabetic nephropathy	1 (11.1)	3 (23.1)
Congenital disease	1 (11.1)	1 (7.7)
Other specified disease	0 (0)	1 (7.7)
Unknown	5 (55.6)	4 (30.8)
Graft characteristics		
Age donor	50 (38–63)	49 (27–75)
HLA-A mismatch	0 (0–1)	1 (0–2)
HLA-B mismatch	1 (0–2)	0 (0–2)
HLA-DR mismatch	0 (0–1)	1 (0–1)
Deceased donor	8 (88.9)	13 (100)
Cold ischemia time, hours	14.2 (2.3–22.3)	15.5 (11.6–27.4)
Induction therapy*		
None	2 (22.2)	0 (0)
Anti-IL-2 antibodies	5 (55.6)	10 (76.9)
Anti-thymocyte globulin	0 (0)	3 (23.1)
Alemtuzumab	2 (22.2)	0 (0)
Plasmapheresis	0 (0)	2 (15.4)
Maintenance therapy		
Cyclosporin	3 (33.3)	9 (69.2)
Tacrolimus	5 (55.6)	4 (30.8)
Mycophenolate mofetil/mycophenolic acid	3 (33.3)	9 (69.2)
Azathioprine	0 (0)	0 (0)
Rapamycine	0 (0)	0 (0)
Belatacept	1 (11.1)	0 (0)
Sotrastaurin	1 (11.1)	0 (0)
Steroids	7 (77.8)	12 (92.3)
Clinical events < 6 months post-transplantation		
Hydronephrosis	2 (22.2)	5 (38.5)
BKV nephritis	0 (0)	0 (0)
Urinary tract infection	0 (0)	4 (30.8)
Sepsis or other severe infection	0 (0)	0 (0)
Graft function		
Serum creatinine, µmol/l	178.0 (101–293)	157.0 (116–383)
Serum creatinine, µmol/l at 6 months	146.0 (107–364)	154 (98–860)
Proteinuria, g/l	0.0 (0.0–0.08)	0.0 (0.0–0.15)
Proteinuria, g/l at 6-months	0.0 (0.0–0.07)	0.0 (0.0–0.08)
eGFR (CKD-EPI), ml/min/1.73 m^2^	36.0 (15–55)	35.0 (14–47)
eGFR (CKD-EPI), ml/min/1.73 m^2^ at 6-months	44.0 (11–58)	33.0 (5–79)
Banff lesion scores		
Total inflammation (ti)	1 (0–2)	1 (0–1)
Inflammation in non-scarred parenchyma (i)	0 (0–1)	0 (0–1)
Inflammation in scarred parenchyma (i-IFTA)	2 (0–3)	2 (0–3)
Interstitial fibrosis (ci)	0 (0–1)	0 (0–1)
Tubular atrophy (ct)	0 (0–1)	1 (0–1)
Banff lesion scores at 6 months		
Total inflammation (ti)	1 (0–2)	1 (0–3)
Inflammation in non-scarred parenchyma (i)	0 (0–1)	0 (0–3)
Inflammation in scarred parenchyma (i-IFTA)*	1 (0–3)	3 (1–3)
Interstitial fibrosis (ci)	0 (0–1)	1 (0–2)
Tubular atrophy (ct)	1 (0–1)	1 (0–2)
IFTA percentages		
IFTA 6 weeks	9.7 (0–30)	7.5 (0.17–22.5)
IFTA 6 months**	5.0 (1.67–33.33)	25.0 (12.5–68.3)
ΔIFTA 6 weeks to 6-months**	1.0 (−12.5–5.0)	19.0 (11.5–61.7)

The median (minimum–maximum value) or occurrences (percentages or minimum–maximum value) are reported.

*BMI* body mass index, *HLA* human leukocyte antigen, *Il-2* interleukin 2, *BKV* BK virus, *eGFR* estimated glomerular filtration rate.

*p < 0.05; **p < 0.001.

### IFTA assessment

The extent of interstitial fibrosis (ci) and tubular atrophy (ct) (IFTA) at 6 weeks and 6 months, expressed using the Banff lesion grading system [24] was acquired from the pathology report. To assess the relationship between early inflammatory infiltrates and IFTA development in more detail, all PAS-stained slides were digitized for re-examination using a Pannoramic 250 Flash II digital slide scanner (3DHistech, Hungary) with a 20× objective at a resolution of 0.24 μm/pixel. The PAS WSI of both time points (6 weeks and 6 months) were scored for the extent of IFTA (percentage of surface area, with 10% intervals) by three kidney pathologists. The mean IFTA scores of the pathologists were used as a final score to calculate the change in IFTA between 6 weeks and 6 months post-transplantation. Patients were stratified by absolute increase in IFTA score of 10% or more (*n* = 13) and no or <10% increase of IFTA (*n* = 9) (Table 1). Recipient characteristics, donor characteristics, and Banff ci, ct, ti, i and i-IFTA lesion scores (obtained from the pathology report) are listed in Table 1 for both patient groups. Significant differences between patient groups were assessed using the independent samples Mann–Whitney U test or Fisher’s exact test and are displayed in Table 1.

In addition, the Banff lesion scores were compared between time points using Wilcoxon signed ranks test. This revealed significant differences between 6 weeks and 6 months biopsies for Banff categories ti (*p* = 0.017), ci (*p* = 0.004), and ct (*p* = 0.011).

### Multiplex TSA staining

We performed multiplex IHC using mTSA to visualize multiple cell markers in the 6 weeks biopsies. After incubation with a primary and secondary antibody, the tissue was treated with fluorescently labeled tyramide. The horse-radish peroxidase from the secondary antibody catalyzes the formation of active tyramide radicals. The tyramide radicals covalently bind to the tyrosine residues on the antigen. This permanent binding allowed for heat-induced removal of the primary–secondary antibody complex, while preserving the fluorescent tyramide deposit [25]. This enabled the subsequent successive incubation with further antibodies from the same species against the target antigens.

mTSA was performed on two consecutive slides from the 6 weeks surveillance biopsies. We developed two mTSA panels to assess the inflammatory infiltrate and peritubular capillary extent in our patient groups. Panel I existed of anti-CD3, CD4, CD8, CD20, CD68, and CD34 antibodies. Panel II was used to investigate the T-helper cell and macrophage polarization by using anti-CD4, Tbet, GATA3, CD68, and CD163 antibody. Antibody specifications, dilutions, and orders of staining are listed in Supplementary Table 1. All slides were deparaffinized in xylene, dehydrated in 95% ethanol, washed in tap water, and boiled for epitope retrieval in 10x diluted tris-borate-EDTA (TBE 10x, 0658, VWR Life Sciences, U.S.) buffer. After cooling down, the slides were washed in 3% hydrogen peroxidase solution for endogenous peroxidase blocking and washed with tris-buffered saline buffer with 0.05% Tween 20 (822184, Merck KGaA, Germany) (TBS-T). Protein blocking was performed using TBS-T with 1% bovine serum albumin (BSA) (mTSA step 1). Primary antibodies were incubated for 1 h at room temperature, or overnight at four degrees Celsius (mTSA step 2). After washing in TBS-T, the slides were incubated with an HRP-conjugated secondary antibody (Poly-HRP-GAMs/Rb IgG, VWRKDPVO999HRP, Immunologic, The Netherlands) for 30 min at room temperature (mTSA step 3). Next, TSA was performed using the Opal TSA fluorophores from an Opal 7-color Manual IHC Kit (NEL811001KT, Akoya Biosciences, U.S.) (mTSA step 4) (fluorophores and their corresponding antibodies are listed in Supplementary Table 1). The antibody-TSA complex was removed with a boiling cycle in TBE buffer (mTSA step 5). mTSA steps 1–5 were repeated until the slides were stained with all antibodies from the concerning panel. The slides were covered with fluoromount-G with DAPI (00-4959-52, Thermo Fisher, U.S.).

### Multiplex TSA validation

Repeated boiling cycles can affect the target epitope affinity. Some antibodies show a weaker staining pattern after the tissue is boiled multiple times, other antibodies need more boiling cycles to reach the optimum staining intensity, and others are not affected at all. We assessed this effect for all antibodies using chromogenic IHC on FFPE control tonsil tissue. For every tested antibody (*n* = 9), six sections were cut (4 μm thick). All slides were deparaffinized in xylene, dehydrated in 95% ethanol, washed in tap water, and boiled for epitope retrieval in 10x diluted TBE (boiling cycle one). After cooling down, one slide per tested antibody was stored in phosphate-buffered saline (PBS). The remaining slides were boiled again. This cycle was repeated five times. All slides were subsequently washed in 3% hydrogen peroxidase solution and followed by rinsing in PBS. Primary antibodies (Supplementary Table 1) were incubated for 1 h at room temperature. After incubation, the slides were washed in PBS. Slides stained with anti-CD68, Tbet, and GATA3 antibody required an additional incubation with post-antibody blocking (PAB) for 15 min (VWRKDPVB blocking, Immunologic, The Netherlands). After incubation, the slides were washed in PBS and incubated with an HRP-conjugated secondary antibody (following PAB VWRKDPVB110HRP, Immunologic, The Netherlands, for others see secondary antibody Supplementary Table 1). Visualization was performed using 3,3′-diaminobenzidine (DAB) (Bright-DAB, VWRKBS04, Immunologic, The Netherlands). The results are visualized in Supplementary Fig. 1. Based on these results, we determined the optimal antibody order for the mTSA experiments, as listed in Supplementary Table 1.

If epitopes of interest are co-localized, the tyramide deposits can interfere with each other. To test for this steric inhibition, we used tonsil control tissue slides and stained these with our mTSA panels. The antibody expression in the mTSA was compared to that in single-stained slides, which went through the same number of boiling cycles. We did not observe differences in staining patterns between the single- and multiplex-stained slides (examples included from panel I, Supplementary Figs. 2 and 3).

All primary antibodies in the mTSA were used in the same dilution that was used for chromogenic IHC. The intensity of the fluorescent signal was optimized by adjusting the TSA solution dilutions.

### Multiplex TSA imaging

Multispectral imaging was performed using a Vectra Polaris Imaging System (CLS143455, Akoya Biosciences, U.S.) with a 20x objective, at a resolution of 0.49 μm per pixel, and using DAPI, FITC, CY3, Texas Red, and Cy5 spectral cubes. The Vectra system allows manual selection of regions for multispectral acquisition, which are subsequently divided by the system into tiles (Fig. 1.1). The spectra of autofluorescence and all Opal TSA fluorophores were prerecorded in a spectral “library” using the Inform Advanced Image Analysis Software 2.4.6. (Akoya Biosciences, U.S.). The spectral library enabled decomposing the multiplex tile into multiple single tiles representing the contribution of each fluorophore (“unmixing”). This resulted in monochrome, multi-channeled tiles, each channel corresponding to a single fluorophore and thus, antibody (Fig. 1.2).

**Fig. 1:**
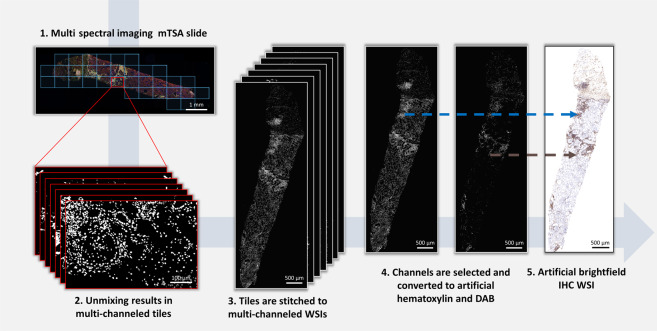
Conversion of an mTSA-stained slide to an artificial brightfield IHC WSI. The mTSA slide was multi spectrally imaged on the Vectra system, resulting in multispectral tiles (1). The tiles were unmixed by the Inform software, leading to multi-channeled tiles where each channel represents one marker (2). The tiles were subsequently stitched into a multi-channeled WSI (3). In this example, the channels representing DAPI and CD4 were selected be combined in one WSI (4). Stain vectors acquired in previous studies were used to artificially color the DAPI signal blue (hematoxylin) and the CD4 signal brown (DAB), resulting in an artificial brightfield IHC WSI (5).

### Conversion to artificial brightfield IHC

Based on stored coordinates, the tiles were stitched to create a multi-channel WSI using a custom python script (Fig. 1.3). The channels representing the DAPI signal (*I*_DAPI_) and the channels representing one of the antibodies (*I*_IHC_) were converted to artificial hematoxylin and DAB staining, respectively (Figs. 1.4 and 1.5). Based on known chromatic hematoxylin and DAB C*x*,C*y* coordinates after hue-saturation-density (HSD) transform, stain vectors were acquired in previous studies [26, 27]. These stain vectors were used to calculate the red-green-blue values for the artificial brightfield IHC (Fig. 1.5), as:

$$R = 255 \cdot e^{ - \left( {I_{{\mathrm{DAPI}}} \cdot c_{R,{\mathrm{Hem}}} + I_{{\mathrm{IHC}}} \cdot c_{R,{\mathrm{DAB}}}} \right)}$$

with *c*_R,st_ the light absorption of dye st in the red part of the spectrum. Values for B and G were calculated in a similar fashion.

### Image analysis

#### Regions of interest (ROIs)

Regions of interest (ROIs) were annotated for every case in the cohort using the automated slide analysis platform software (ASAP; version 1.9, available as open-source software on GitHub). These ROIs comprised of cortical tubulointerstitium, thus excluding the capsule, glomeruli, and arteries. Since inflammation in renal subcapsular regions is considered non-specific in transplant pathology, the biopsies in this study were primarily analyzed excluding the subcapsular region (defined as 400 µm below the capsule). Secondarily, we repeated the analyses including the subcapsular region. Visual examples of the ROIs are included in Supplementary Fig. 4.

#### Lymphocyte detection CNN I

The artificial brightfield IHC images representing CD3, CD4, CD8, and CD20 staining were analyzed using an existing CNN with a U-Net architecture [22, 28]. This network was specifically designed for the detection of cytoplasmatic lymphocyte markers in IHC. CNN performance can be expressed in precision, recall, and an F1-score, where:

$${\mathrm{Precision}} = \frac{ {\mathrm{True}}\;{\mathrm{positive}}\;{\mathrm{detections}}\,{\mathrm{(TP)}}}{ {\mathrm{True}}\;{\mathrm{positive}}\;{\mathrm{detections}}\;\left( {\mathrm{TP}} \right) + {\mathrm{False}}\;{\mathrm{positive}}\;{\mathrm{detections}}\,{\mathrm{(FP)}}}$$

$${\mathrm{Recall}} = \frac{ {\mathrm{True}}\;{\mathrm{positive}}\;{\mathrm{detections}}\,{\mathrm{(TP)}}}{ {\mathrm{True}}\;{\mathrm{positive}}\;{\mathrm{detections}}\left( {\mathrm{TP}} \right) + {\mathrm{False}}\;{\mathrm{negative}}\;{\mathrm{detections}}\,{\mathrm{(FN)}}}$$

$${\mathrm{F1}} = 2 \cdot \frac{ {\mathrm{Precision}} \cdot {\mathrm{Recall}}}{ {\mathrm{Precision}} + {\mathrm{Recall}}}$$

The CNN achieved a precision of 0.76, a recall of 0.79, and a F1-score of 0.78 on the test set that was used in the original paper, comprising of traditional IHC WSI. Detection of individual positive cells requires thresholding the CNN output, followed by postprocessing. Because the CD3 staining in the mTSA panel was stronger compared to CD4, CD8, and CD20, a lower object detection threshold was used for the latter three (0.4) and the original object detection threshold for CD3 (0.7). To assess the CNN performance on the artificial brightfield IHC WSIs in this study, four artificial brightfield IHC WSI (CD8 and CD20 from two patients) were used as a test set in this study. Dot annotations (*n* = 1115) were generated using ASAP software. After applying the network, precision, recall, and F1-score were calculated to assess the CNN performance. Detections were considered true positive if they were found within 4 µm (average lymphocyte diameter) from a ground truth annotation. When two detections were found within a 4 µm range, only the detection that was closest to the annotation was considered true positive. Subsequently, lymphocyte detection CNN I was used for the analysis of all artificial brightfield IHC WSI representing cytoplasmatic lymphocyte markers (CD3, CD4, CD8, and CD20).

#### Lymphocyte detection CNN II

The analysis of artificial brightfield IHC WSI with nuclear staining patterns (as presented by Tbet and GATA3) required training, validation, and testing of a new CNN. For this purpose, nine slides were cut from kidney, tonsil, and appendix FFPE control tissue. These slides were IHC-stained with anti-Tbet (clone 4B10, 14-5825-82, Thermo Fisher Scientific, U.S.) and anti-GATA3 (clone L50-823, CM-405B, Biocare Medical, The Netherlands) antibody. The slides were digitized using a Pannoramic 250 Flash II digital slide scanner at a resolution of 0.12 μm/pixel. Two observers produced 5726 dot annotations across different regions using ASAP software. Annotations from five slides were used for training a U-Net architecture CNN using patches of 256 × 256 pixels with a pixel size of 0.49 μm/pixel. Two WSI were used for validation of the CNN and for determining the object detection threshold (0.4). The CNN performance on traditional IHC WSI was assessed on a withheld test set of two IHC WSI. CNN performance on artificial brightfield IHC WSI was assessed on a secondary test set comprising of four artificial brightfield IHC WSI (Tbet and GATA3 from two patients) with 1082 dot annotations. Precision, recall, and F1-score were calculated to assess the performance on both test sets. Detections were considered true positive if they were found within 4 µm from a ground truth annotation. When two detections were found within a 4 µm range, only the detection that was closest to the annotation was considered true positive. Subsequently, lymphocyte detection CNN II was used for the analysis of all artificial brightfield IHC WSIs representing nuclear (lymphocyte) markers (Tbet and GATA3).

#### Macrophage detection CNN

In contrast to lymphocyte detection, the identification of individual macrophages is not unequivocal. Especially in clustered scenes, a significant level of observer variability can be expected. Therefore, a much larger number of cases and human annotations were used to train a dedicated, third CNN for the detection of CD68^+^ and CD163^+^ macrophages. IHC-stained slides (*n* = 111) from native and transplant kidney tissue were collected. IHC stainings were performed using anti-CD68 (clone PG-M1, GA61361-2, Dako Omnis, Denmark or clone KP1, M0876, Dako, Denmark) or anti-CD163 (clone MRQ-26, or 10D6, NCL-L-CD163, Leica Biosystems, U.K) antibody. The IHC slides were digitized using a Pannoramic 250 Flash II digital slide scanner or an Aperio AT2 Slide Scanner (Leica Biosystems, Wetzlar, Germany) at a resolution of 0.24 or 0.25 μm/pixel, respectively. Four observers produced 37,709 dot annotations across multiple ROIs in the WSIs, using a protocol for macrophage annotation, which was agreed upon after initial pilot experiments. The annotations from 101 slides were used for training of a YoloV2 architecture CNN [29]. Yolo is specifically suited for tasks aimed at detection tasks. The network, consisting of seven convolutional layers, was trained on patches of 256 × 256 pixels extracted at a resolution of 0.98 μm/pixel with bounding boxes of 21 μm (based on average macrophage size). Ten WSI were used for validation of the CNN and for determining the object detection threshold (0.45) and non-maximum suppression parameters (0.05). The CNN performance on traditional IHC WSI was assessed on a withheld test set of ten IHC WSI. CNN performance on artificial brightfield IHC WSI was assessed on a secondary test set comprising of four artificial brightfield IHC WSI (CD68 and CD163 from two patients) with 1033 dot annotations. Precision, recall, and F1-scores were calculated to assess the performance on both test sets. Detections were considered true positive if they were found within 21 µm (average macrophage diameter) from a ground truth annotation. When more detections were found within a 21 µm range, only the detection that was closest to the annotation was considered true positive. Subsequently, the macrophage detection CNN was used for the analysis of all artificial brightfield IHC WSI representing macrophage markers (CD68 and CD163).

#### Double positivity

Positivity of cells for two markers (double positivity) was assessed by determining the number of pixels between cell detections in the different channels. If the distance between two lymphocyte detections was <4 µm, the cell was considered double-positive. For macrophages, this was set to <21 µm. This was used to assess CD3^+^CD4^+^, CD3^+^CD8^+^, CD4^+^Tbet^+^, CD4^+^GATA3^+^, and CD68^+^CD163^+^ cells. Cell numbers were calculated inside the ROIs, and cell densities were based on cell count and the area of the annotated ROI.

#### Spatial relationships

Automated cell detection in WSI allows the investigation of spatial relationships between cells. The mean shortest distance was determined (in regions excluding the subcapsular region) for CD68^+^ cells and CD3^+^, CD3^+^CD8^+^, and CD20^+^ cells in the WSI of panel I for both patient groups, and between CD163^+^ cells and CD4^+^, CD4^+^Tbet^+^, and CD4^+^GATA3^+^ in the WSI for both patient groups.

#### Peritubular capillary extent

In order to assess peritubular capillary extent, unmixed WSIs representing the CD34 channel were analyzed in Fiji (ImageJ version 2.0.0, U.S., macros and plugins: “Open and Duplicate”, “ASAP ROI Reader”) [30]. Positive pixels were determined via automatic thresholding and subsequently expressed as the percentage of the total number of pixels inside the ROI.

### Statistical analysis

The densities of the following cell populations were calculated in the 6 weeks biopsies: T-lymphocytes (CD3^+^), cytotoxic T-lymphocytes (CD3^+^CD8^+^), B-lymphocytes (CD20^+^), macrophages (CD68^+^, panels I and II), polarized macrophages (CD68^+^CD163^+^, CD163^+^), T-helper 1 lymphocytes (CD4^+^Tbet^+^), and T-helper 2 lymphocytes (CD4^+^GATA3^+^). Spearman’s correlation coefficients were calculated to assess if a correlation was present between T-helper 1 and T-helper 2 lymphocyte density (CD4^+^Tbet^+^, CD4^+^GATA3^+^) and polarized macrophage density (either CD68^+^CD163^+^ or CD163^+^). We observed CD68 signal (fluorophore 540 nm) in the artificial CD4 (fluorophore 520 nm) IHCs of panel I. Therefore, we additionally report the cell densities for CD3^+^CD8^−^ cells. To assess differences between patient groups with different IFTA outcomes, we report median, minimum, and maximum cell density values per group. Significant differences in cell density and peritubular capillary extent (defined as the CD34-positive pixel percentage) between groups were assessed using the Mann–Whitney’s U test for independent samples. Whether patients with different IFTA outcome show significantly different CD3^+^CD8^−^/CD3^+^CD8^+^ cell ratios, was assessed using a *t*-test for independent samples. Differences between patient groups in spatial relationships of CD68^+^ and CD163^+^ cells with other immune cells were assessed for significance using the Mann–Whitney’s U test for independent samples.

## Results

### CNN-based detection of IHC positive cells

**Fig. 2:**
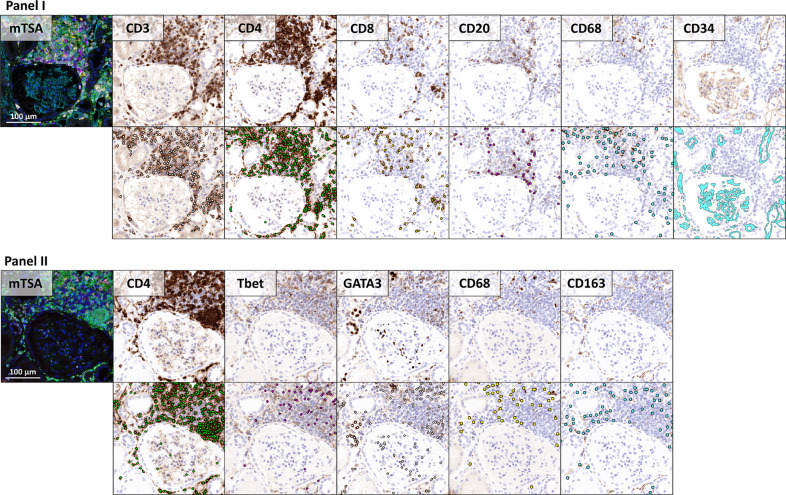
Regions from two mTSA-stained slides, displaying the multiplex IHC and the artificial brightfield representation for every antibody. First and third row: multiplex IHC (left) and artificial brightfield images for every antibody (brown) combined with DAPI (blue). Second and bottom row: cell detections performed by the CNNs (lymphocytes and macrophages, filled circles) and segmented regions through image processing (capillaries, CD34, filled shapes).

In order to apply existing CNNs, which were originally developed for brightfield microscopy, mTSA fluorescence images were transformed to artificial brightfield images. Examples of mTSA-stained regions with their corresponding artificial brightfield IHC images are included in Fig. 2. An example of an artificial brightfield IHC WSI is demonstrated in Supplementary Fig. 4. The multi-resolution WSIs could be opened and viewed in digital slide viewing software such as ASAP and Aperio ImageScope [v12.4.3.5008]. As visualized in Fig. 2, the artificial brightfield IHC WSI were suitable for automated analysis by CNNs that were originally developed for traditional IHC WSI.

Three CNNs were used for the quantitative assessment of inflammatory cells in the 6 weeks mTSA-stained transplant biopsies: for lymphocyte detection with cytoplasmic (CNN I) and nuclear (CNN II) IHC staining and for macrophage detection. Table 2 shows CNN performance (precision, recall, and F1-scores) for hold-out sets of both DAB-stained IHC WSIs and artificial brightfield IHC WSIs. CNN performance was typically as good as, or better than the baseline CNN described previously (with an F1-score of 0.78), which was shown to possess performance comparable to experienced manual observers [22]. Whereas the lymphocyte detection CNN II showed somewhat reduced performance on virtual brightfield images as compared to the real DAB images (on which the CNN was trained), the opposite was observed for the CNN for macrophage detection.

**Table 2:** Performance of the CNNs that were used for quantitative assessment of inflammatory infiltrates in this study.

	Traditional IHC WSI			Artificial brightfield IHC WSI		
	Precision	Recall	F1	Precision	Recall	F1
Lymphocyte detection CNN I [22]	0.76^a^	0.79^a^	0.78^a^	0.92	0.73	0.81
Lymphocyte detection CNN II	0.81	0.88	0.84	0.71	0.84	0.77
Macrophage detection CNN	0.79	0.75	0.77	0.93	0.74	0.82

^a^Data from original research paper [22].

**Fig. 3:**
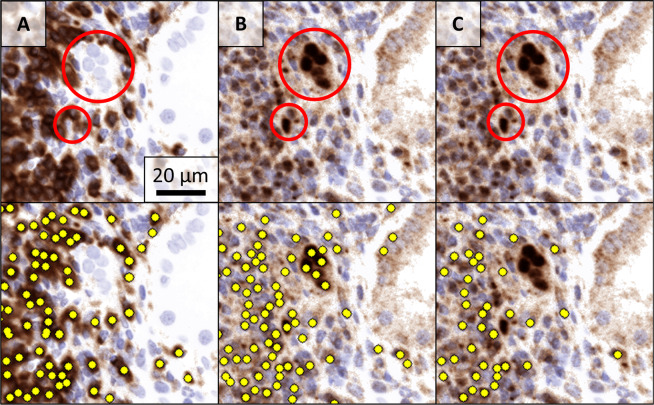
Using distance of cell detections to include CD4^+^GATA3^+^ cells and exclude GATA3^+^ epithelial cells from the analysis. Column **A**: artificial IHC representing CD4 without (top) and with (bottom) cell detections. The epithelial cells (red circle) are negative for CD4. Column **B**: artificial IHC representing GATA3 without (top) and with (bottom) cell detections. The epithelial cells (red circle) are positive for GATA3 and detected by the neural network. Column **C**: artificial IHC representing GATA3 without (top) and with (bottom) cell detections closer than eight pixels to a CD4 cell detection. The epithelial cells (red circle) are removed from the cell detections.

An example of successful automatic double positivity assessment is included in Fig. 3.

### Correlation of different cell types

The strongest correlation was observed between CD4^+^GATA3^+^ cell density and CD163^+^ cell density (Spearman’s coefficient 0.75, *p* < 0.001) in the 6 weeks biopsy (Supplementary Fig. 5A). This correlation was weaker between CD4^+^Tbet^+^ cell density and CD163^+^ cell density (Spearman’s coefficient 0.61, *p* < 0.01) (Supplementary Fig. 5B). When limiting the cell population to double-positive macrophages (CD68^+^CD163^+^), Spearman’s correlation coefficient was 0.65 (*p* < 0.01) with CD4^+^GATA3^+^ cells and 0.66 (*p* < 0.01) with CD4^+^Tbet^+^ cells (Supplementary Fig. 5C, D). Including the subcapsular region in the analyses did not alter the results.

### Comparison of inflammatory infiltrates between patients progressing to IFTA versus non-IFTA

Patients progressing to IFTA at 6 months displayed significantly higher CD163^+^ cell densities in the biopsies taken 6 weeks after transplantation (median 505 cells/mm^2^) versus patients that did not progress to IFTA (median 370 cells/mm^2^; *p* = 0.043) (Table 3). Inclusion of the subcapsular region resulted in a slight reduction of this effect (*p* = 0.051). CD68 and CD4 were used in both panels. Slides stained with mTSA panel I showed more CD68 positivity than the slides stained with mTSA panel II. CD4 cell density is higher in mTSA panel II compared to mTSA panel I (Table 3).

**Table 3:** Median CD34^+^ pixel percentages, cell densities cells/mm^2^ (min–max) and mean cell ratios (standard deviation) in the cortical tubulointerstitium of the 6 weeks biopsies, excluding the subcortical region.

	ΔIFTA < 10% (*n* = 9)	ΔIFTA ≥ 10% (*n* = 13)	*p* value
Panel I			
CD34^+^	7.77 (6.30–12.35)	8.17 (6.62–11.07)	0.74
CD3^+^	413 (90–861)	303 (93–905)	0.65
CD3^+^CD4^+^	70 (8–186)	39 (2–300)	0.56
CD3^+^CD8^+^	23 (7–235)	32 (8–268)	0.19
CD3^+^CD8^−^	296 (79–821)	221 (81–827)	0.70
CD20^+^	6 (0–59)	8 (2–211)	0.21
CD68^+^	203 (90–532)	328 (142–578)	0.07
Panel II			
CD4^+^	88 (13–680)	197 (27–1215)	0.39
CD4^+^Tbet^+^	3 (0–58)	6 (0–102)	0.29
CD4^+^GATA3^+^	11 (0–241)	51 (1–249)	0.24
CD68^+^	92 (8–459)	72 (27–351)	0.90
CD163^+^	370 (105–625)	505 (112–781)	0.04
CD68^+^CD163^+^	74 (8–368)	64 (24–315)	1
Cell ratios			
CD3^+^CD8^−^/CD3^+^CD8^+^	17.47 (9.05)	9.80 (7.55)	0.04

Peritubular capillary extent was similar in 6 weeks biopsies of DGF patients with different IFTA outcomes (Table 3), both when excluding (*p* = 0.74) and including (*p* = 0.90) the subcapsular region from/in the analysis.

Assessment of CD3^+^CD8^−^/CD3^+^CD8^+^ cell ratios showed a significantly higher ratio in patients with <10% IFTA development 6 months post-transplantation (ratio of 17.5) than in patients with ≥10% IFTA development (ratio of 9.80; *p* = 0.043) (Table 3).

**Fig. 4:**
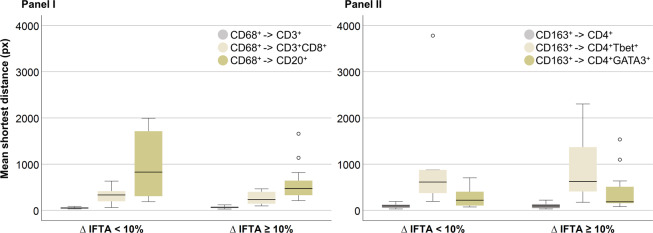
Mean shortest cell distances. Boxplots representing the mean shortest distance (measured in pixels (px)) from CD68^+^ cells (panel I) and CD163^+^ cells (panel II) to other immune cells, based on analyses excluding the subcapsular region, according to ∆IFTA percentages 6 weeks and 6 months post-transplantation.

The mean shortest distance from CD68^+^ cells to CD3^+^, CD3^+^CD8^+^, and CD20^+^ cells (panel I) and from CD163^+^ cells to CD4^+^, CD4^+^Tbet^+^, and CD4^+^GATA3^+^ cells (panel II) did not differ significantly between patient groups. The results are visualized in Fig. 4.

## Discussion

In this study, we developed a method for the accurate and objective quantification of inflammatory cell infiltrates in graft biopsies of kidney transplant patients with DGF that circumvents extensive serial cutting of kidney biopsy material. For this purpose, we combined multiplex IHC, tyramide signal amplification, multispectral imaging, and quantification by CNNs. We were the first to convert tiled multispectral data to one single artificial chromogenic image per cell marker, facilitating WSI analysis and application of CNNs designed for brightfield IHC. We designed two new CNNs for the detection of nuclear-stained lymphocytes and macrophages and demonstrated the generalizability of CNNs developed on traditional IHC WSI to artificial brightfield IHC WSI. The applicability of our method was demonstrated by using the quantitative results obtained by the CNNs to study correlations of the inflammatory microenvironment in 6 weeks biopsies of DGF patients with the development of IFTA 6 months post-transplantation.

We used a commercially available manual staining kit for multiplex IHC to visualize immune cells and peritubular capillaries in surveillance biopsies obtained 6 weeks post-transplantation. The multiplex staining procedure consisted of multiple washing, incubation, and tissue boiling steps and involves several reagent solutions. Extensive method validations and quality controls are therefore of great importance, and use of specific antibodies that yield consistent staining intensity are recommended. Despite the performed validation steps, macrophage-like staining patterns were seen in the CD4 channels of slides from mTSA panel I and II. CD4 and CD68 staining cycles were not performed consecutively, thus this phenomenon could not be caused by incomplete stripping of the CD68 antibody (Supplementary Table 1). Although rare occurrences of macrophage dual-positivity with CD4 has been described [31], a more plausible explanation lays in the proximity of the fluorophores’ emission spectra that were used for CD4 (520 nm) and CD68 (540 nm) visualization, both covered by the FITC filter cube of the fluorescence microscope. This can cause “bleeding” of the strong CD68 signal into the CD4 channel. Much of this signal was excluded from analysis in panel I, because only CD4^+^ cells that were double-positive with CD3 were used for general T-helper cell analysis. Nonetheless, we decided to indirectly assess general T-helper cells as well, using CD3^+^CD8^−^ as a replacement. In panel II, CD4 was solely used in combination with Tbet and GATA3, limiting the risk for the use of false positive detections.

Lower CD68 positivity was observed in panel II compared to panel I. We hypothesize that this is the result of steric inhibition by tyramide deposit belonging to CD163 (“umbrella effect”) [32]. We observed significantly more CD163-positive cells in the studied cohort than in tonsil tissue that was used to check for steric inhibition, possibly explaining why this effect was not discovered during validation.

Multiplex IHC has been combined with multispectral imaging for the examination of the tumor microenvironment in several oncology studies, and recently also for the analysis of kidney allograft rejection [33–35]. To extract the contribution of all markers in mTSA slides, sections are imaged with a Vectra system or a similar fluorescence microscope with a multispectral set up. After recording a low-magnification overview image, the Vectra system divides the tissue into tiles and automatically scans the tiles multi spectrally. This results in image tiles with multiple contributing spectra. Because the spectra of the single fluorophores are known from the prerecorded spectral “library”, it is possible to decompose the multiplex tiles into multiple single tiles representing the contribution of each fluorophore (“unmixing”). In most studies, the unmixed images are subsequently analyzed with commercial software. In many cases, these programs do not support WSI analysis, have difficulty analyzing clustered cells and are often not resilient to artifacts and staining variations. Converting the unmixed tiles to artificial brightfield IHC WSIs, enabled us to apply an existing CNN specifically designed for lymphocyte detection in IHC [22] (referred to as lymphocyte detection CNN I). This network can detect individual and clustered lymphocytes with high accuracy while being resilient to background staining (Fig. 2, CD3). In addition, we trained two new CNNs for the detection of cells with nuclear staining patterns (Tbet, GATA3) (lymphocyte detection CNN II) and for the detection of macrophages. Macrophages are notoriously difficult to detect due to their scattered staining pattern. The macrophage detection CNN was therefore trained using the annotations of four different experts. Prior to making the annotations, multiple meetings were planned where the criteria for annotating macrophages were discussed and assessed. This resulted in a network that can detect macrophages in a reproducible fashion while being robust for non-specific staining (Table 2, Fig. 2, and Supplementary Fig. 6). To our knowledge, this is the first algorithm for macrophage detection in scanned histopathological sections. We tested the performance of all three networks on a test set comprised of traditional IHC WSI (similar to those used during training) and on a secondary test set that consisted of artificial brightfield IHC WSI, generated from the multi spectrally recorded images. All CNNs show very good performance on the primary test sets and similar F1-scores on the secondary test sets. The performance metrics of lymphocyte detection CNN I were calculated on normal tissue, artifacts, and cell clusters. The artificial brightfield IHC of the secondary test set contained no tissue artefacts and less cell clusters. This can explain the overall better performance of this network on the secondary test set. The macrophage detection CNN was trained and tested on annotations from four different annotators. While annotation criteria were particularly discussed, variations in annotation style were observed nonetheless. The CNN’s sensitivity is therefore probably somewhere in the middle of the annotation style extremes. The annotations for the secondary test set were generated by one annotator, seemingly matching the CNN sensitivity.

Using the described CNNs allowed us to investigate the inflammatory infiltrate with unprecedented accuracy in a unique series of rigorously selected early surveillance biopsies of transplant patients with DGF.

Unfortunately, multiple samples had to be excluded from analysis, mostly due to insufficient residual tissue after diagnostic work-up. Even with the limited size of the data set, we found significantly higher CD163^+^ cell densities in biopsies of DGF patients who progressed to the development of IFTA, which is in line with the potentially pro-fibrotic role of these cells [11]. While the observed trend was consistent with published data, we could not confirm the detrimental effect of early presence of CD68^+^ macrophages that has been previously reported for other kidney transplant patient groups [7, 36, 37]. We found a positive correlation between the densities of CD4^+^GATA3^+^ cells and CD163^+^ cells, which might confirm the contribution of T-helper 2 lymphocytes toward a pro-fibrotic microenvironment. While no new predictive biomarkers for IFTA development in DGF patients were discovered in this study, we successfully developed methods for the accurate, reproducible, and scalable assessment of inflammatory infiltrate in sparse tissue such as transplant biopsies. These methods are valuable for future quantitative studies on inflammation in histopathological tissue.

## Supplementary information

Supplemental material

## Data Availability

Collaboration requests involving the use of data presented in this study can be addressed to the corresponding author (jeroen.vanderlaak@radboudumc.nl) or FF (Feuerhake.Friedrich@mh-hannover.de).
