# Supplementary material for: Quantitative assessment of inflammatory infiltrates in kidney transplant biopsies using multiplex tyramide signal amplification and deep learning
Source: Lab Invest. 2021 May 18;101(8):970–82. doi: 10.1038/s41374-021-00601-w (PMC8292146; doi:10.1038/s41374-021-00601-w)
Supplement: Supplementary file 1 — Supplemental material [file 41374_2021_601_MOESM1_ESM.pdf]

SUPPLEMENTARY TABLE 1. Specifications of antibodies, TSA-solutions, and multiplex staining order.

Panel I					
Position	*Primary antibody*	*Dilution*	*Secondary antibody*	*TSA fluorophore*	*TSA dilution*
1	CD34 (QBEnd/10) mouse monoclonal antibody, ILM1343, Immunologic, The Netherlands	1:400	Poly-HRP-GAMs/Rb IgG, VWRKDPVO999HRP, Immunologic, The Netherlands	TSA 690, FP1497A, Perkin Elmer, U.S.	1:100
2	CD4 (EP204) rabbit monoclonal antibody, 104R-24, Cell Marque, U.S.	1:200	Poly-HRP-GAMs/Rb IgG, VWRKDPVO999HRP, Immunologic, The Netherlands	TSA 520, FP1487A, Perkin Elmer, U.S.	1:100
3	CD8 (C8/144B) mouse monoclonal antibody, M 7103, Dako, Denmark	1:80	Poly-HRP-GAMs/Rb IgG, VWRKDPVO999HRP, Immunologic, The Netherlands	TSA 570, FP1488A, Perkin Elmer, U.S.	1:100
4	CD3 (SP7) rabbit monoclonal antibody, RM-9107-S, Thermo Scientific, U.S.	1:40	Poly-HRP-GAMs/Rb IgG, VWRKDPVO999HRP, Immunologic, The Netherlands	TSA 650, FP1496A, Perkin Elmer, U.S.	1:200
5	CD68 (PG-M1), mouse monoclonal antibody, M 0876, Dako, Denmark	1:40	Poly-HRP-GAMs/Rb IgG, VWRKDPVO999HRP, Immunologic, The Netherlands	TSA 540, FP1494A, Perkin Elmer, U.S.	1:100
6	CD20 (L26), mouse monoclonal antibody MS-340-S, Thermo Scientific, U.S.	1:300	Poly-HRP-GAMs/Rb IgG, VWRKDPVO999HRP, Immunologic, The Netherlands	TSA 620, FP1495A, Perkin Elmer, U.S.	1:100
**Panel II**					
1	CD68 (PG-M1), mouse monoclonal antibody, M 0876, Dako, Denmark	1:40	Poly-HRP-GAMs/Rb IgG, VWRKDPVO999HRP, Immunologic, The Netherlands	TSA 540, FP1494A, Perkin Elmer, U.S.	1:100
2	CD163 (10D6) mouse monoclonal antibody, NCL-L-CD163, Leica Biosystems, U.K.	1:500	Poly-HRP-GAMs/Rb IgG, VWRKDPVO999HRP, Immunologic, The Netherlands	TSA 620, FP1495A, Perkin Elmer, U.S.	1:200
3	Tbet (4B10) mouse monoclonal antibody, 14-5825-82, Thermo Fisher Scientific, U.S.	1:200	Poly-HRP-GAMs/Rb IgG, VWRKDPVO999HRP, Immunologic, The Netherlands	TSA 570, FP1488A, Perkin Elmer, U.S.	1:100
4	CD4 (EP204) rabbit monoclonal antibody, 104R-24, Cell Marque, U.S.	1:200	Poly-HRP-GAMs/Rb IgG, VWRKDPVO999HRP, Immunologic, The Netherlands	TSA 520, FP1487A, Perkin Elmer, U.S.	1:100
5	GATA3 (L50-823) mouse monoclonal antibody, CM 405 B, Biocare Medical, The Netherlands	1:100	Poly-HRP-GAMs/Rb IgG, VWRKDPVO999HRP, Immunologic, The Netherlands	TSA 650, FP1496A, Perkin Elmer, U.S.	1:100

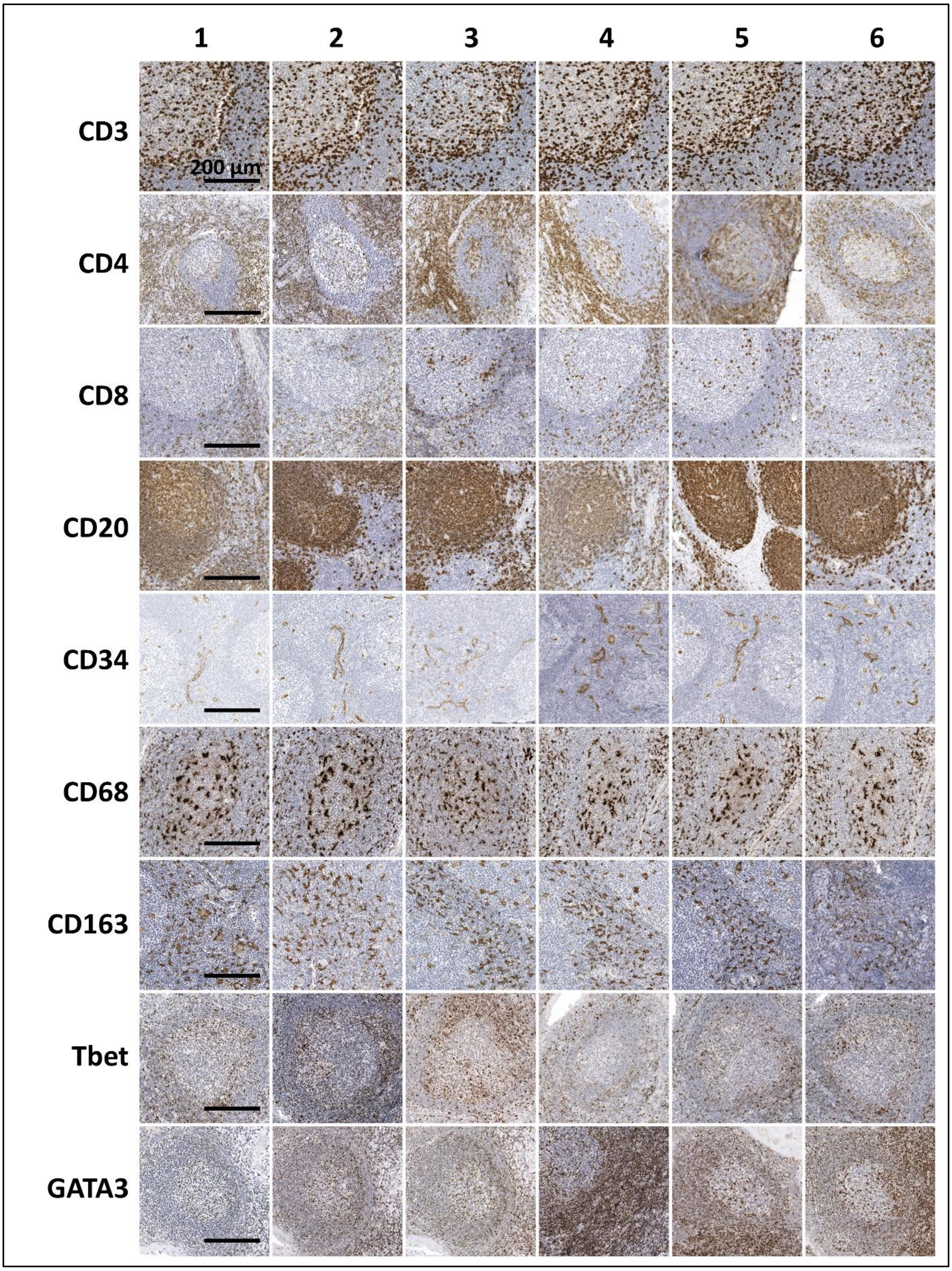

SUPPLEMENTARY FIGURE 1. Epitope sensitivity for boiling cycles. Vertical: antibody type. Horizontal: number of boiling cycles. GATA3 shows increased expression after multiple boiling cycles, the expression of the other antibodies is not affected.

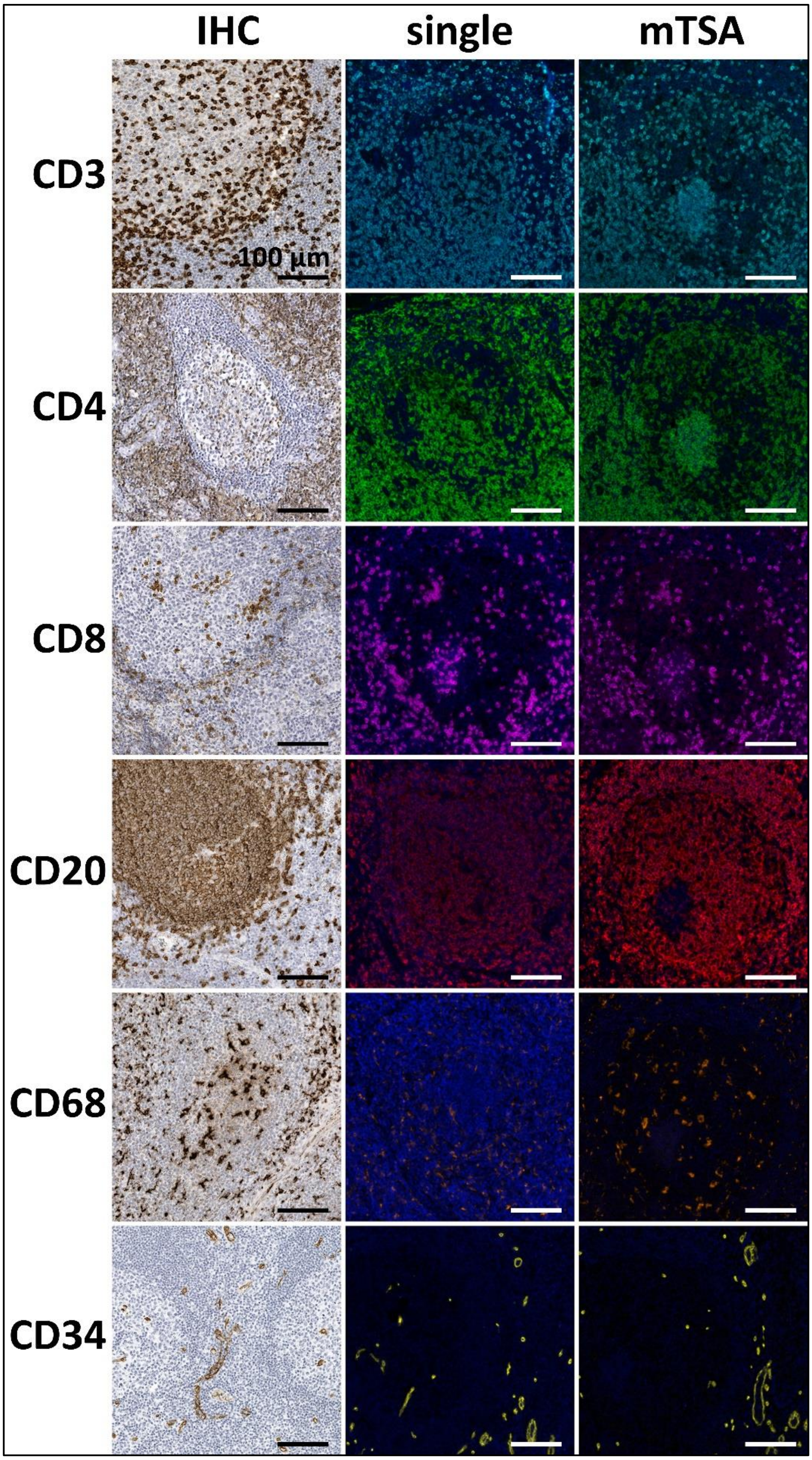

SUPPLEMENTARY FIGURE 2. Validation steric inhibition panel I. The mTSA signal was not lower than that in in single staining. No steric inhibition was present.

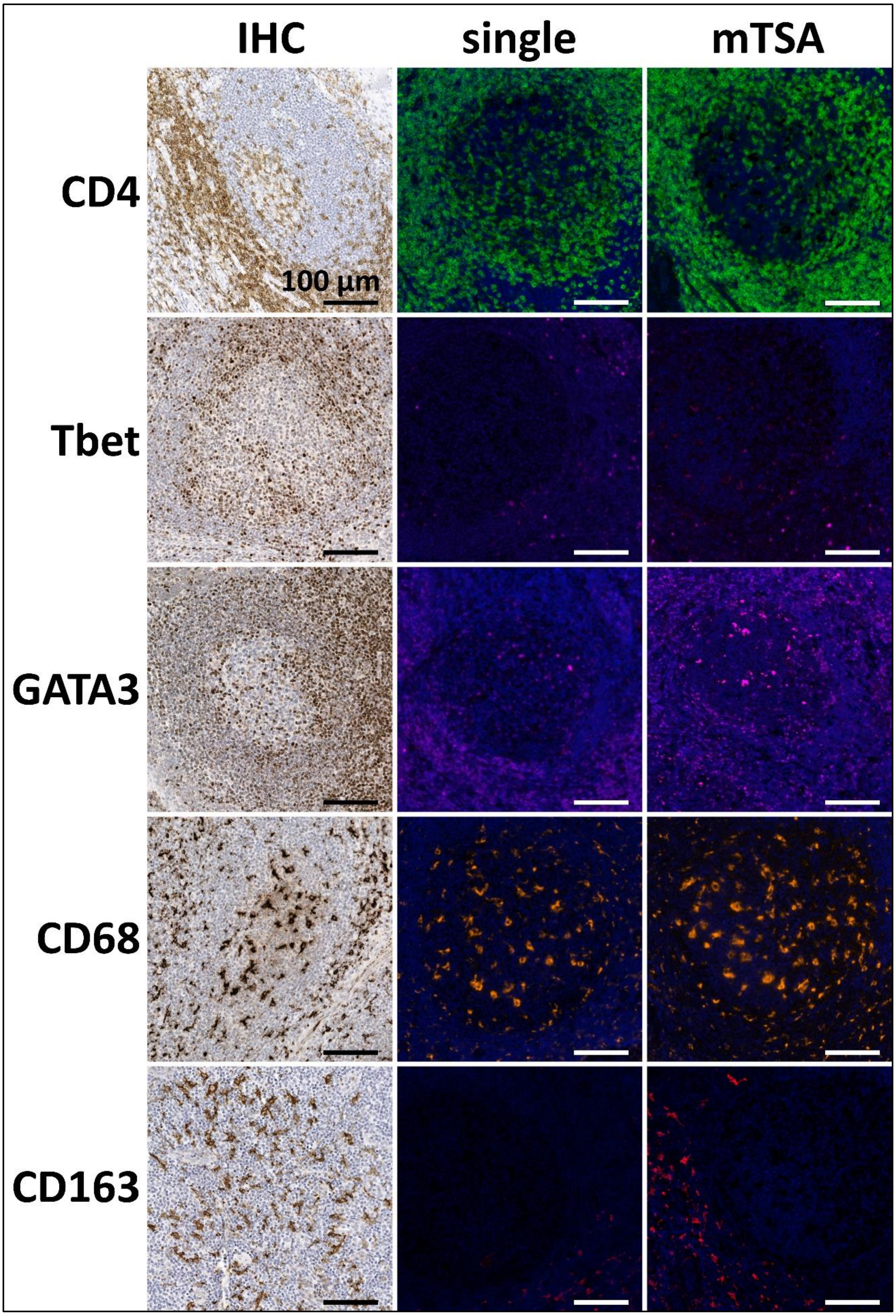

SUPPLEMENTARY FIGURE 3. Validation steric inhibition panel II. The mTSA signal was not lower than that in in single staining. No steric inhibition was present.

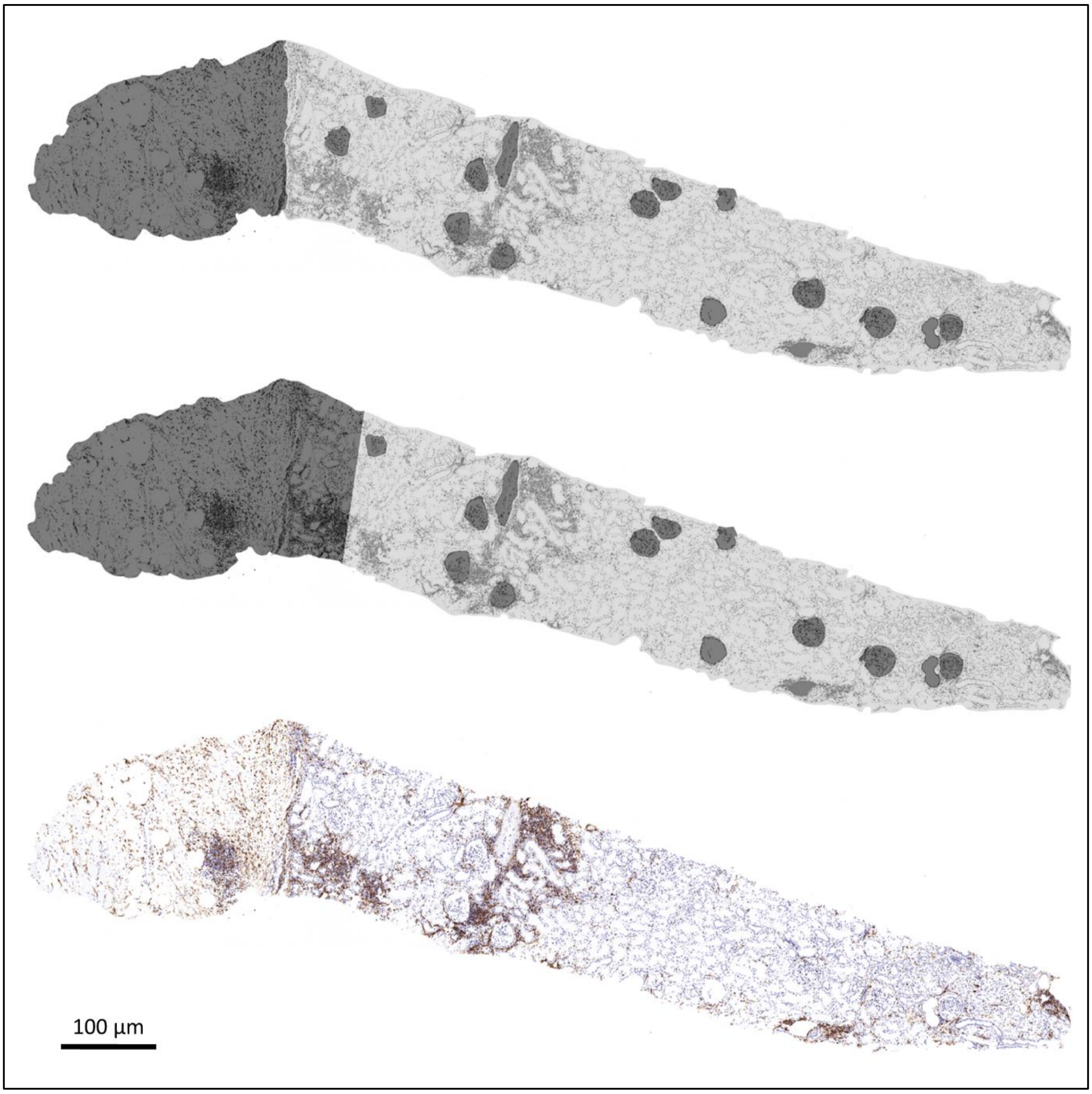

SUPPLEMENTARY FIGURE 4. Examples of regions of interest in artificial brightfield whole-slide image. Dark grey: excluded regions, light grey: regions included in the analyses (cortical tubule interstitium). Top: mask representing a region of interest including the subcapsular region. Middle: mask representing a region of interest excluding the subcapsular region. Bottom: artificial brightfield whole-slide image without mask.

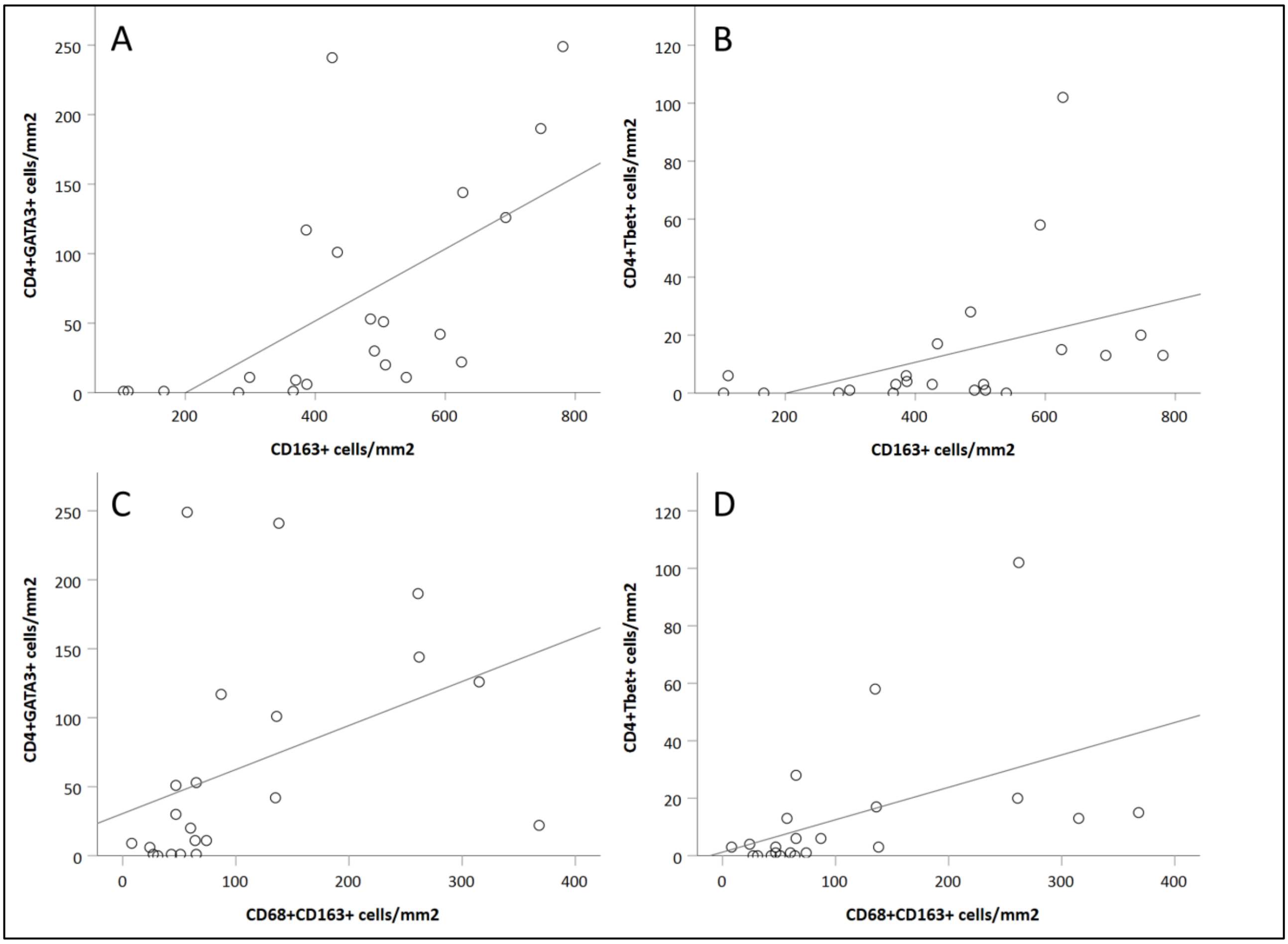

SUPPLEMENTARY FIGURE 5. Scatterplots correlation different cell types.

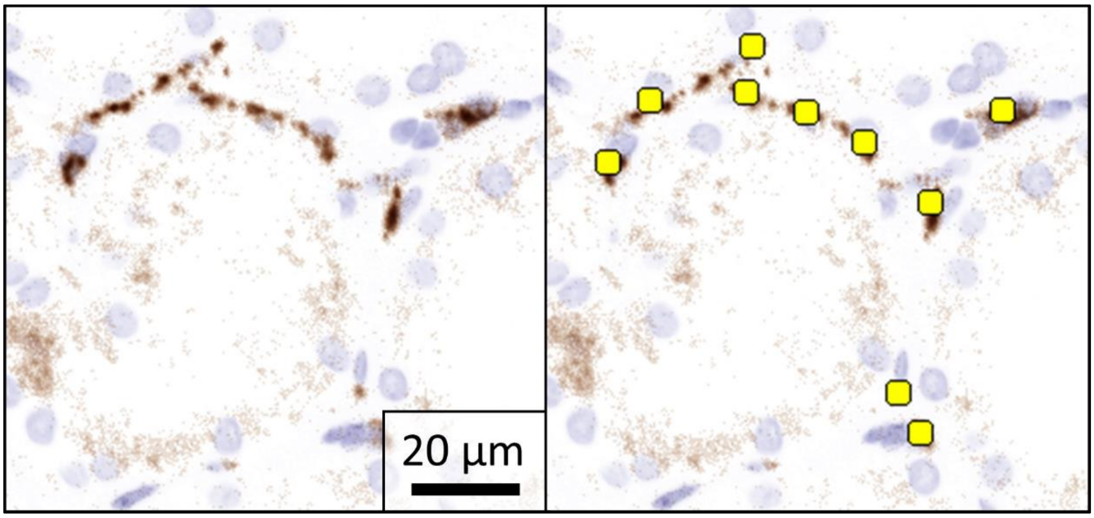

SUPPLEMENTARY FIGURE 6. Visual example macrophage detection network on high magnification. Left: artificial brightfield IHC representing CD68. Right: cell detections by the neural network.
